# The 14-3-3γ isoform binds to and regulates the localization of endoplasmic reticulum (ER) membrane protein TMCC3 for the reticular network of the ER

**DOI:** 10.1016/j.jbc.2022.102813

**Published:** 2022-12-20

**Authors:** Saihas Suhda, Yasunori Yamamoto, Sindhu Wisesa, Risa Sada, Toshiaki Sakisaka

**Affiliations:** Division of Membrane Dynamics, Department of Physiology and Cell Biology, Kobe University School of Medicine, Kobe, Japan

**Keywords:** endoplasmic reticulum, protein phosphorylation, 14-3-3 protein, membrane protein, protein–protein interaction, ER, endoplasmic reticulum, SPG, spastic paraplegias, TMCC3, transmembrane and coiled-coil domain family 3, PDI, protein disulfide isomerase

## Abstract

The reticular network of the endoplasmic reticulum (ER) is formed by connecting ER tubules through three-way junctions and undergoes constant remodeling through formation and loss of the three-way junctions. Transmembrane and coiled-coil domain family 3 (TMCC3), an ER membrane protein localizing at three-way junctions, has been shown to positively regulate formation of the reticular ER network. However, elements that negatively regulate TMCC3 localization have not been characterized. In this study, we report that 14-3-3γ, a phospho-serine/phospho-threonine-binding protein involved in various signal transduction pathways, is a negative regulator of TMCC3. We demonstrate that overexpression of 14-3-3γ reduced localization of TMCC3 to three-way junctions and decreased the number of three-way junctions. TMCC3 bound to 14-3-3γ through the N terminus and had deduced 14-3-3 binding motifs. Additionally, we determined that a TMCC3 mutant substituting alanine for serine to be phosphorylated in the binding motif reduced binding to 14-3-3γ. The TMCC3 mutant was more prone than wildtype TMCC3 to localize at three-way junctions in the cells overexpressing 14-3-3γ. Furthermore, the TMCC3 mutant rescued the ER sheet expansion caused by TMCC3 knockdown less than wild-type TMCC3. Taken together, these results indicate that 14-3-3γ binding negatively regulates localization of TMCC3 to the three-way junctions for the proper reticular ER network, implying that the negative regulation of TMCC3 by 14-3-3γ would underlie remodeling of the reticular network of the ER.

The endoplasmic reticulum (ER) is the largest organelle playing essential roles in various fundamental cellular processes including protein synthesis and quality control, lipid synthesis, membrane traffic, calcium storage, autophagy, organelle biogenesis, and detoxification of harmful substances (1, 2, 3, 4, 5, 6, 7, 8, 9). The ER is the continuous membrane system composed of two building blocks, ER sheets and ER tubules (10, 11, 12, 13). While the ER sheets are predominantly localized at the perinuclear region, the ER tubules are interconnected by three-way junctions, leading to formation of the reticular network of the ER tubules throughout the cytoplasm (10, 11, 12, 13). It has been shown that the reticular ER network is shaped by four classes of the ER membrane proteins conserved from yeasts to mammals, reticulons, REEPs/DP1/Yop1, atlastins, and lunapark (10, 12, 13, 14, 15, 16, 17, 18). Reticulons and REEPs/DP1/Yop1 are targeted to the ER membrane (15, 19, 20) and constrict the ER membrane by inserting their wedge-like transmembrane domains into the outer leaflet of the ER membrane (21, 22), leading to generation and stabilization of the ER tubules and the edges of the ER sheets, both of which are characterized by relatively high membrane curvature in cross-section (11, 13, 14, 16, 18, 21, 22). The ER tubules are interconnected by atlastins, dynamin-like GTPases (17, 18, 23, 24, 25). Atlastins are anchored on the ER tubules and form a bridge between the tip of an ER tubule and the side of another one (17, 18, 25, 26, 27). Upon GTP hydrolysis, atlastins drive homotypic membrane fusion between the two ER tubules, leading to generation of the three-way junction (17, 18, 24, 25, 26, 27). Lunapark is then recruited to the nascent three-way junctions and sits on their concave edges, leading to stabilization of the three-way junctions (28, 29, 30, 31).

Mammalian cells have been shown to employ some additional ER membrane proteins in order to shape the ER membrane. In addition to reticulons and REEPs/DP1/Yop1, mammalian cells employ Arl6IP1 for formation of the ER tubules and the ER sheets (32). We have previously demonstrated that Arl6IP1 is the ER membrane protein that has the wedge-like transmembrane domains homologous to reticulons and generates and stabilizes the ER tubules and the edges of the ER sheets in the same manner as reticulons (32). Protrudin has been shown to regulate formation of the reticular ER network by binding to reticulons, REEPs, and atlastins (33, 34). Arl6IP1 and protrudin are also known as SPG (spastic paraplegias) 61 and SPG33, respectively, because hereditary spastic paraplegias, a group of inherited neurological disorders whose pathology is characterized by axonal degeneration of corticospinal motor neurons, is caused by pathogenic mutations in *Arl6IP1* and *Protrudin* genes (35, 36, 37, 38). TMEM170A also regulates formation of the reticular ER network by binding to reticulons (39). Furthermore, we have recently demonstrated that TMCC3 (transmembrane and coiled-coil domain family 3) is involved in shaping the ER membrane in mammalian cells (40). TMCC3 is a higher eukaryote-specific ER membrane protein composed of the large N-terminal cytoplasmic region containing two coiled-coil domains and the short C-terminal region containing two transmembrane domains (40). TMCC3 specifically localizes to three-way junctions and binds to atlastins through the C-terminal transmembrane domains, regulating formation of the reticular ER network (40). TMCC3 has been shown to be associated with formation of the membrane contact sites between ER and endosomes (41) and maintenance of breast cancer stem cells (42). These evidences underscore the biological importance of the mammalian-specific mechanisms to shape the ER membrane.

The reticular network of the ER tubules is not static but undergoes constant remodeling through regulation of the three-way junctions. While new three-way junctions are frequently generated, a subpopulation of the existing three-way junctions slides along the intersecting ER tubules, leading to loss of the ER polygons (ring closure) (28, 29, 43, 44, 45). Lunapark has been shown to regulate sliding of the existing three-way junctions and loss of the ER polygons (28, 29). On the other hand, the molecular mechanism of how three-way junction formation is regulated for the constant remodeling still remains unclear. One potential regulator for this would be TMCC3, because we have recently demonstrated that TMCC3 is upstream of atlastins (40). While TMCC3 knockdown decreases the number of three-way junctions and expands the ER sheets, overexpression of altastin-2, but not overexpression of lunapark, rescues the phenotypes of the TMCC3-knockdown cells (40), indicating that TMCC3 regulates formation of the three-way junctions by enhancing the atlastin activity. Given that TMCC3 localizes to three-way junctions independently of binding to atlastins (40), molecular mechanisms that regulates localization of TMCC3 to three-way junctions would play important roles in formation of the three-way junction during the constant remodeling. We have shown that the first coiled-coil domain of TMCC3 positively regulates localization of TMCC3 to the three-way junctions (40). However, it still remains unknown how localization of TMCC3 to the three-way junctions is negatively regulated.

An earlier study has identified 14-3-3 proteins as proteins that bind to TMCC3 (46). The human 14-3-3 family is composed of seven family members (β, γ, ε, η, σ, τ, and ζ) (47), each of which exists as dimer in the cytosol (47, 48, 49). 14-3-3 proteins have been widely accepted as phosphoserine/phosphothreonine-binding proteins (47, 50, 51, 52, 53). 14-3-3 proteins interact with phosphorylated serine or threonine within the consensus binding motif, RXXpS/TXP, where pS/T represents phosphorylated serine or threonine (54, 55). A diverse array of proteins, including signaling proteins, enzymes, transcription factors, and proto-oncogene proteins is known to have the consensus binding motif and be targeted by 14-3-3 proteins (51, 52, 53). The 14-3-3 proteins bind to these target proteins in phosphorylation-dependent manner, which in turn sequester the target proteins in the cytosol, leading to regulation of various cellular processes including cell growth, cell survival, metabolism, apoptosis, cellular stress responses, protein trafficking, and cancer progression (51, 52, 53). However, it remains unknown whether 14-3-3 proteins are involved in regulation of the ER morphology, in particular, whether binding of 14-3-3 proteins to TMCC3 is involved in regulation of the reticular ER network.

In this study, we characterize the interaction between 14-3-3γ and TMCC3 in the context of regulation of the ER morphology and demonstrate that 14-3-3γ binds to TMCC3 through phosphorylated serine in the consensus binding motif, negatively regulating localization of TMCC3 to the three-way junctions. Given that TMCC3 is the upstream regulator of atlastins, these results imply that the negative regulation of TMCC3 by 14-3-3γ would underlie remodeling of the three-way junction formation.

## Results

### Overexpression of 14-3-3γ reduces localization of TMCC3 to three-way junctions

**Figure 1:**
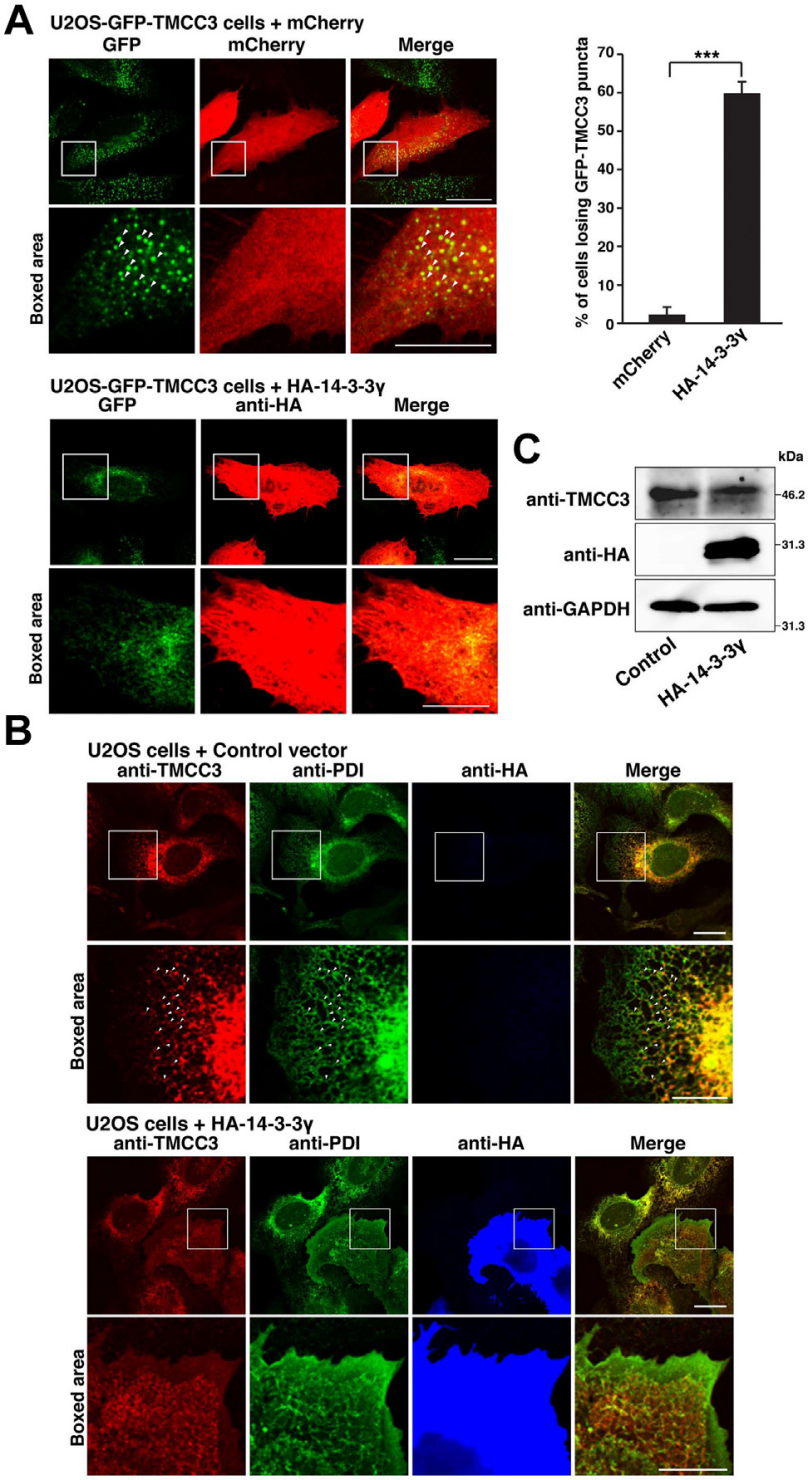
**Overexpression of 14-3-3γ reduces localization of TMCC3 to three-way junctions**. *A*, overexpression of 14-3-3γ reduces localization of GFP-TMCC3 to three-way junctions. HA-14-3-3γ or mCherry was transfected into U2OS-GFP-TMCC3 cells. Six hours after transfection, the cells were fixed and permeabilized. The cells transfected with HA-14-3-3γ were subjected to immunostaining with the anti-HA mAb. GFP-TMCC3 and mCherry were detected by GFP fluorescence and mCherry fluorescence, respectively. Representative images of three independent experiments are shown in the *left panels*. Scale bars, 20 μm. The *boxed area* are enlarged to highlight GFP-TMCC3 puncta and shown below each image. *Arrowheads* indicate the representatives of GFP-TMCC3 puncta. Scale bars, 10 μm. Sixty transfected cells were randomly chosen, and the number of cells losing GFP-TMCC3 puncta was counted. The ratio of the cells losing GFP-TMCC3 puncta to the transfected cells is expressed as a percentage and shown in the *right graph*. The error bars represent SD of three independent experiments. Statistical analysis was performed using Student’s *t* test. ∗∗∗*p* < 0.001. *B*, overexpression of 14-3-3γ reduces localization of endogenous TMCC3 to three-way junctions. HA-14-3-3γ or a control vector was transfected into U2OS cells, followed by immunostaining with the anti-TMCC3 pAb, the anti-PDI mAb, and the anti-HA mAb. Representative images of three independent experiments are shown. Scale bars, 20 μm. The *boxed areas* are enlarged to highlight localization of TMCC3 and shown below each image. The *red* and *green* images are merged in the rightmost column. *Arrowheads* indicate localization of TMCC3 at three-way junctions. Scale bars, 10 μm. *C*, effect of overexpression of 14-3-3γ on the protein level of TMCC3. HA-14-3-3γ or the control vector was transfected into U2OS cells. The total cell lysates were subjected to immunoblotting with the anti-TMCC3 pAb, the anti-HA mAb, and the anti-GAPDH. TMCC3, transmembrane and coiled-coil domain family 3.

While the earlier study has demonstrated that 14-3-3 proteins bound to TMCC3 (46), there is no study characterizing the interaction between 14-3-3 proteins and TMCC3 in the context of regulation of the ER morphology. In this study, we set out to examine the effect of 14-3-3 proteins on localization of TMCC3 to three-way junctions. While human 14-3-3 family is composed of seven isoforms, the earlier study did not clarify whether TMCC3 bound to 14-3-3 proteins in isoform-dependent manner or not (46). On the other hand, BioGRID (56), the database for protein and genetic interactions, shows that 14-3-3γ and 14-3-3θ are identified as TMCC3-binding proteins. We, therefore, employed 14-3-3γ as a representative of 14-3-3 proteins in this study. We established U2OS cell lines (U2OS-GFP-TMCC3 cells) stably expressing GFP-tagged TMCC3 (GFP-TMCC3). The U2OS-GFP-TMCC3 cells were immunostained for protein disulfide isomerase (PDI), an ER marker protein, and GFP fluorescence was compared with PDI staining. Consistent with our previous finding that TMCC3 localized at three-way junctions, GFP-TMCC3 showed puncta-like localization indicative of three-way junctions (Fig. S1). HA-tagged 14-3-3γ (HA-14-3-3γ) or mCherry was then transfected into the U2OS-GFP-TMCC3 cells, followed by immunostaining with the anti-HA mAb. About 60% of the cells overexpressing HA-14-3-3γ significantly lost the GFP-TMCC3 puncta indicative of three-way junctions, whereas almost all of the cells overexpressing mCherry retained the puncta (Fig. 1*A*). We next examined whether overexpression of HA-14-3-3γ affected localization of endogenous TMCC3. HA-14-3-3γ or a control vector was transfected into U2OS cells, followed by immunostaining with the anti-TMCC3 pAb, the anti-PDI mAb, and the anti-HA mAb. While puncta of endogenous TMCC3 were detected at the three-way junctions in the peripheral ER in the cells transfected with the control vector, the peripheral puncta of endogenous TMCC3 were obviously reduced in the cells overexpressing HA-14-3-3γ (Fig. 1*B*). It is also noted that the PDI staining pattern in the peripheral ER seemed to be affected by overexpression of HA-14-3-3γ to some extent, suggesting that overexpression of 14-3-3γ affected the peripheral ER morphology. These results indicate that overexpression of 14-3-3γ reduced localization of TMCC3 to three-way junctions.

We next examined whether overexpression of 14-3-3γ affected the protein level of TMCC3. HA-14-3-3γ or the control vector was transfected into U2OS cells. The total cell lysates were subjected to immunoblotting with the anti-TMCC3 pAb, the anti-HA mAb, and the anti-GAPDH. Overexpression of HA-14-3-3γ did not change the protein level of endogenous TMCC3 (Fig. 1*C*).

Collectively, these results suggest that 14-3-3 binding changes localization of TMCC3 without affecting the protein level.

### Overexpression of 14-3-3γ decreases the number of three-way junctions

**Figure 2:**
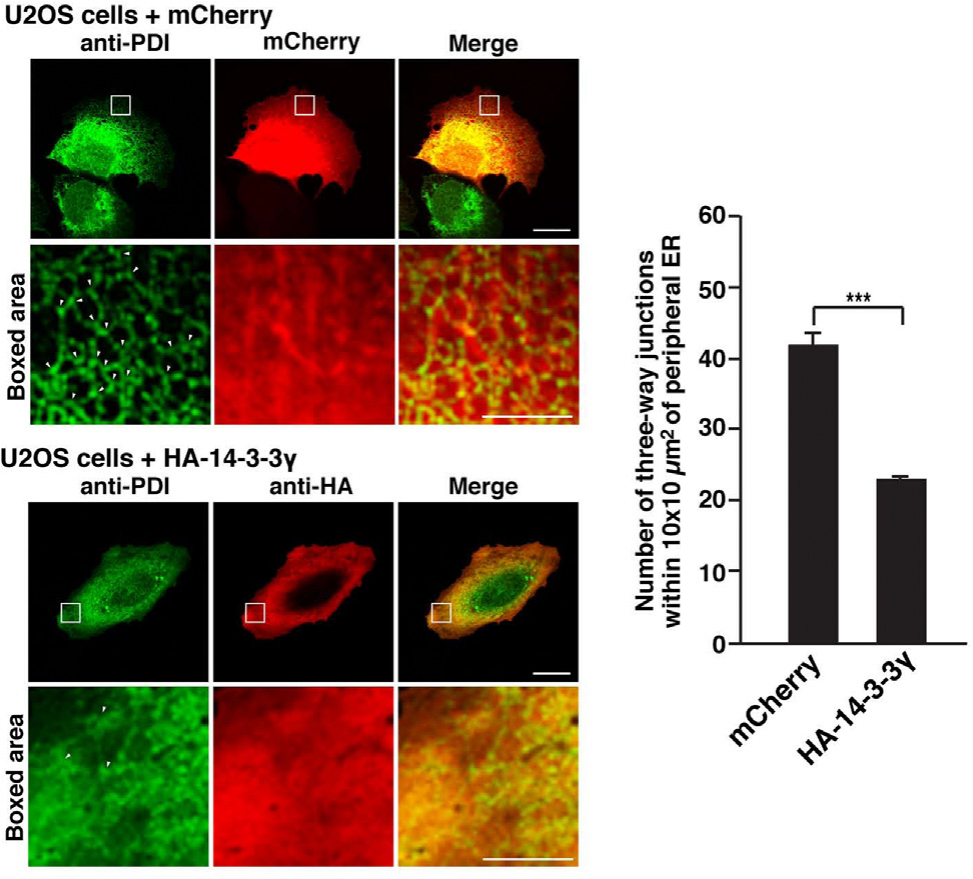
**Overexpression of 14-3-3γ decreases the number of three-way junctions**. HA-14-3-3γ or mCherry was transfected into U2OS cells, followed by immunostaining with the anti-PDI mAb and the anti-HA mAb. mCherry was detected by its fluorescence. Representative images of three independent experiments are shown in the *left panels*. Scale bars, 20 μm. The *boxed areas* represent 10 × 10 μm^2^ of the peripheral ER and enlarged in the *lower panels*. *Arrowheads* indicate representatives of the three-way junctions. Scale bars, 5 μm. Twenty transfected cells were randomly chosen, and the number of three way junctions as detected by PDI staining was counted within 10 × 10 μm^2^ of the peripheral ER. The number of three-way junctions per cell is shown in the *right graph*. The error bars represent SD of three independent experiments. Statistical analysis was performed using Student’s *t* test. ∗∗∗*p* < 0.001. ER, endoplasmic reticulum; PDI, protein disulfide isomerase.

Since TMCC3 is involved in formation of the reticular ER network (40), we reasoned that if 14-3-3γ negatively regulated TMCC3 localization as shown in Fig. 1*B*, overexpression of 14-3-3γ would affect the ER morphology. Indeed, we observed that overexpression of HA-14-3-3γ affected the peripheral ER morphology to some extent as mentioned above. To further assess this, HA-14-3-3γ or mCherry was transfected into U2OS cells, followed by immunostaining with the anti-PDI mAb and the anti-HA mAb. The number of three-way junctions as detected by PDI staining was counted within 10 × 10 μm^2^ of the peripheral ER. In agreement with the above reasoning, the cells overexpressing HA-14-3-3γ significantly decreased the number of three-way junctions relative to the cells overexpressing mCherry (Fig. 2).

To further confirm these results, we also carried out the similar experiments using ER-GFP, GFP fused to the ER signal sequence of calreticulin and KDEL (ER retention signal), as an ER marker. HA-14-3-3γ or the control vector was transfected into U2OS cells, followed by transfection with ER-GFP using the recombinant baculovirus carrying ER-GFP (CellLight ER-GFP, BacMam 2.0, ThermoFisher). The cells were then immunostained with the anti-HA mAb and the anti-GFP mAb. Overexpression of HA-14-3-3γ decreases the reticular staining of ER-GFP and decreased the number of three-way junctions as detected by ER-GFP (Fig. S2), being consistent with the results of Figure 2.

Overall, these results indicate that overexpression of 14-3-3γ decreases the number of peripheral three-way junctions and alters the peripheral ER morphology to some extent.

### TMCC3 binds to 14-3-3γ through the N terminus

**Figure 3:**
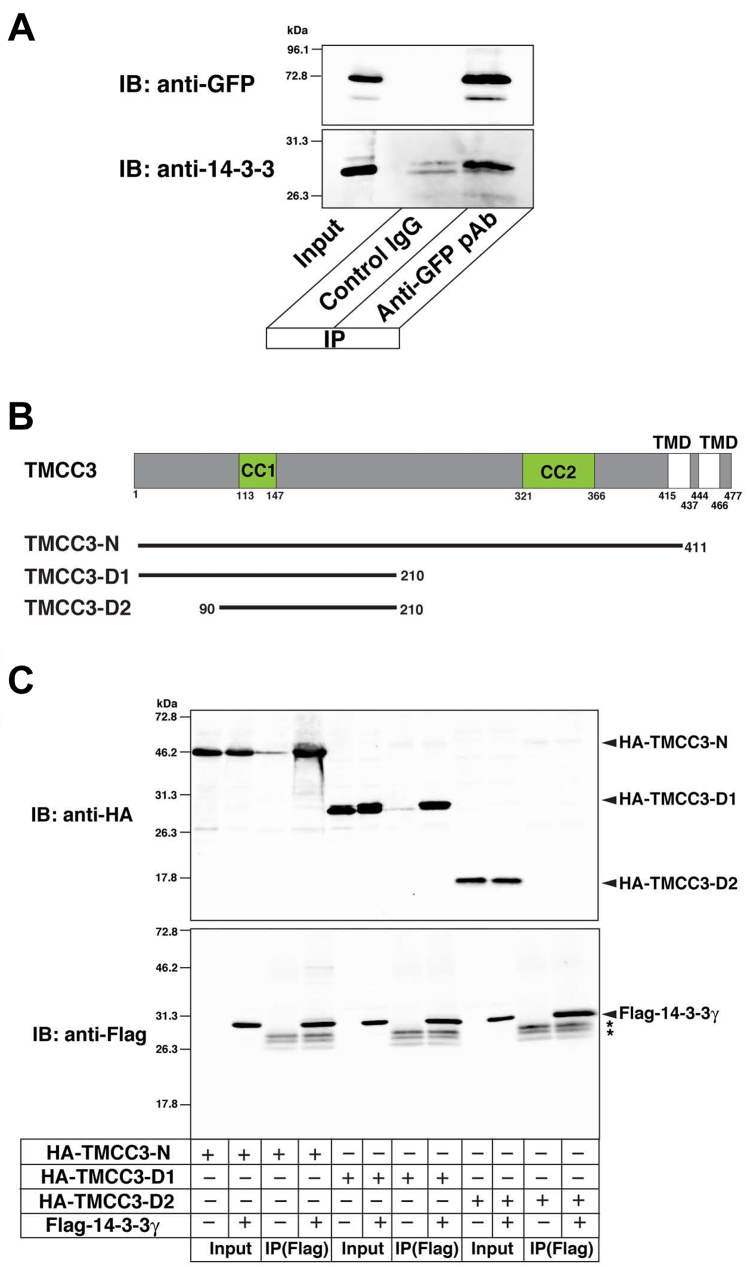
**TMCC3 binds to 14-3-3γ through the N terminus**. *A*, coimmunoprecipitation of GFP-TMCC3 and endogenous 14-3-3 proteins. Triton X-100 extracts of U2OS-GFP-TMCC3 cells were subjected to immunoprecipitation with the rabbit anti-GFP pAb or the rabbit control IgG, followed by immunoblotting with the anti-14-3-3 mAb and the rat anti-GFP mAb. *B*, schematic representation of the deletion mutants of TMCC3-N. TMCC3-D1 was generated by deleting almost C-terminal half from TMCC3-N. TMCC3-D2 was generated by deleting the first 89 amino acids from TMCC3-D1. *C*, the N-terminal 89 aa is responsible for binding to 14-3-3γ. Indicated combinations of HA-TMCC3-N, HA-TMCC3-D1, HA-TMCC3-D2, and Flag-14-3-3γ were transfected into HEK293 cells, followed by immunoprecipitation with the anti-Flag mAb. The samples were immunoblotted with the anti-HA mAb and the anti-Flag pAb. The *asterisks* indicate the nonspecific bands from the light chain of IgG. TMCC3, transmembrane and coiled-coil domain family 3.

We sought to determine which region of TMCC3 was responsible for binding to 14-3-3γ. U2OS-GFP-TMCC3 cells were extracted with 1% Triton X-100 and subjected to immunoprecipitation with the anti-GFP pAb, followed by immunoblotting with the anti-14-3-3 mAb and the anti-GFP mAb. Endogenous 14-3-3 proteins were coimmunoprecipitated with GFP-TMCC3 (Fig. 3*A*), confirming that TMCC3 bound to endogenous 14-3-3 proteins. We next transfected the fragments of TMCC3 with the N-terminal HA tags into HEK293 cells along with FLAG-14-3-3γ, followed by immunoprecipitation with anti-FLAG mAb. The HA-tagged N-terminal fragment encompassing the large cytoplasmic region (HA-TMCC3-N) was coimmunoprecipitated with FLAG-14-3-3γ (Fig. 3, *B* and *C*), indicating that the N-terminal cytoplasmic region had the binding region for 14-3-3γ. To determine the binding region, the deletion mutants of TMCC3-N were generated (Fig. 3*B*) and subjected to immunoprecipitation analysis. Similarly to HA-TMCC-N, the HA-tagged mutant deleting almost C-terminal half from TMCC3-N (HA-TMCC3-D1) was coimmunoprecipitated with FLAG-14-3-3γ (Fig. 3*C*). By contrast, the HA-tagged mutant deleting the first 89 amino acids (aa) from TMCC3-D1 (TMCC3-D2) was not coimmunoprecipitated with FLAG-14-3-3γ. These results indicate that the N-terminal 89 aa is the binding region for 14-3-3γ.

### Phosphorylated serine 15 is required for potent binding to 14-3-3γ

**Figure 4:**
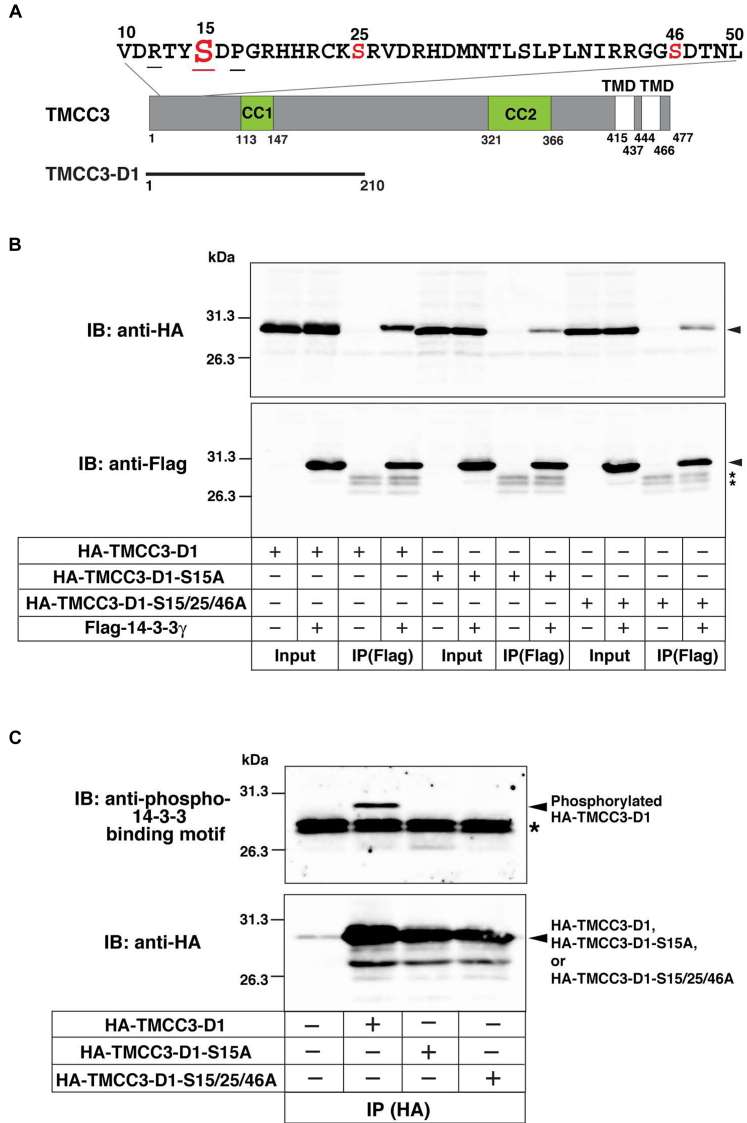
**Phosphorylated serine 15 in the deduced 14-3-3 binding motif is required for potent binding to 14-3-3γ**. *A*, the N-terminal region of TMCC3 highlighting the deduced 14-3-3 binding motifs. Serine residues being the possible phosphorylation sites in three deduced 14-3-3 binding motifs are indicated in *red*. Arginine, serine, and proline residues at −3, 0, and +2 positions, respectively, in the deduced 14-3-3 binding motif predicted with the highest score are underlined. *B*, serine 15 is required for potent binding to 14-3-3γ. Indicated combinations of HA-TMCC3-D1, HA-TMCC3-D1-S15A, HA-TMCC3-D1-S15/25/46A, and Flag-14-3-3γ were transfected into HEK293 cells, followed by immunoprecipitation with the anti-Flag mAb. The samples were immunoblotted with the anti-HA mAb and the anti-Flag pAb. The *arrowheads* indicate the bands of interest. The *asterisks* indicate the nonspecific bands from the light chain of IgG. *C*, serine 15 is phosphorylated. HA-TMCC3-D1, HA-TMCC3-D1-S15A, and HA-TMCC3-D1-S15/25/46A were transfected into HEK293 cells, followed by immunoprecipitation with the anti-HA mAb. The samples were immunoblotted with the anti-phospho-14-3-3 binding motif pAb and the anti-HA pAb. The *asterisk* indicates the nonspecific bands from the light chain of IgG. TMCC3, transmembrane and coiled-coil domain family 3.

14-3-3 proteins are well known to interact with phosphorylated serines or threonines within the consensus binding motif RXXpS/TXP where pS/T represents phosphorylated serine or threonine (54, 55). Therefore, we examined whether TMCC3 had the 14-3-3 binding motif in the N-terminal 89 aa. Full-length aa sequence of mouse TMCC3 was subjected to 14-3-3-Pred, the web server to predict 14-3-3 binding motifs (57). 14-3-3-Pred showed that there were three deduced 14-3-3 binding motifs in the N-terminal 89 aa. Among them, the deduced binding motif at 12 to 17 aa was predicted with the highest score and completely meets the consensus binding motif (Fig. 4*A*). The other two motifs (22–27 aa and 43–48 aa) were predicted with low scores and lacked proline at position +2 in the consensus binding motif. It has been well established that upon substituting alanine for serine to be phosphorylated, the consensus binding motif abolishes or reduces binding to 14-3-3. Therefore, we generated the point mutant of TMCC3-D1 substituting alanine for serine 15 in the deduced binding motif predicted with the highest score (TMCC3-D1-S15A). HA-TMCC3-D1 or HA-tagged TMCC3-D1-S15A (HA-TMCC3-D1-S15A) was transfected into HEK293 cells along with FLAG-14-3-3γ, followed by immunoprecipitation with the anti-FLAG mAb. While HA-TMCC3-D1 was strongly coimmunoprecipitated with FLAG-14-3-3γ, coimmunoprecipitation of HA-TMCC3-D1-S15A was significantly decreased relative to HA-TMCC3-D1 (Fig. 4*B*). These results indicate that serine 15 was required for potent binding to 14-3-3γ. Since TMCC3-D1-S15A still showed weak binding to 14-3-3γ, we further substituted alanines for serine 25 and serine 46 in TMCC3-D1-S15A (TMCC3-D1-S15/25/46A) and performed immunoprecipitation analysis. A small amount of HA-tagged TMCC3-D1-S15/25/46A (HA-TMCC3-D1-S15/25/46A), which was comparable to that of HA-TMCC3-D1-S15A, was coimmunoprecipitated with FLAG-14-3-3γ. This result indicates that the other two deduced binding motifs predicted with the low scores are not involved in binding to 14-3-3γ.

We examined whether serine 15 was phosphorylated. HA-TMCC3-D1, HA-TMCC3-D1-S15A, or TMCC3-D1-S15/25/46A was transfected into HEK293 cells, followed by immunoprecipitation with the anti-HA mAb. The samples were subjected to immunoblotting with the antibody recognizing phosphorylated serine in the consensus 14-3-3 binding motif (anti-phospho-14-3-3 binding motif pAb) and the anti-HA mAb. The anti-phospho-14-3-3 binding motif pAb detected HA-TMCC3-D1 but did not recognize HA-TMCC3-D1-S15A and HA-TMCC3-D1-S15/25/46A (Fig. 4*C*), indicating that serine 15 was phosphorylated. To further validate phosphorylation of serine 15, we carried out the similar experiments in U2OS cells. The anti-phospho-14-3-3 binding motif pAb detected HA-TMCC3-D1 but did not recognize HA-TMCC3-D1-S15A in U2OS cells (Fig. S3).

Collectively, these results indicate that phosphorylated serine 15 in the deduced 14-3-3 binding motif is required for potent binding to 14-3-3γ.

### The TMCC3 mutant substituting alanine for serine 15 is prone to localize at three-way junctions against overexpression of 14-3-3γ

**Figure 5:**
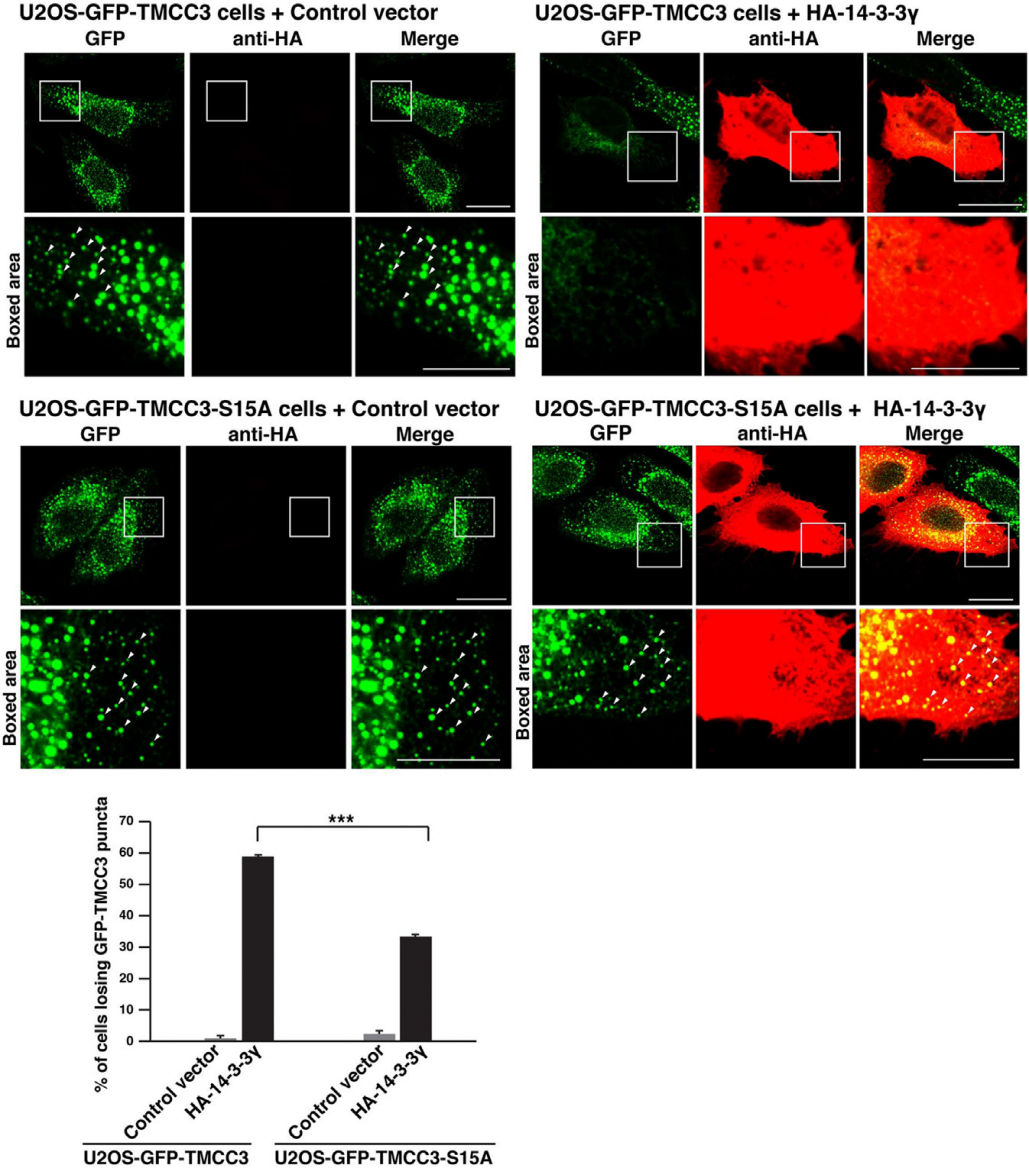
**The TMCC3 mutant substituting alanine for serine 15 is prone to localize at three-way junctions against overexpression of 14-3-3γ**. HA-14-3-3γ or a control vector was transfected into U2OS-GFP-TMCC3 cells and U2OS-GFP-TMCC3-S15A cells. Six hours after transfection, the cells were immunostained with the anti-HA mAb. GFP-TMCC3 and GFP-TMCC3-S15A were detected by GFP fluorescence. Representative images of six independent experiments are shown. Scale bars, 20 μm. The *boxed areas* are enlarged to highlight GFP-TMCC3 puncta and GFP-TMCC3-S15A puncta and shown below each image. *Arrowheads* indicate representatives of GFP-TMCC3 puncta and GFP-TMCC3-S15A puncta. Scale bars, 10 μm. Sixty transfected cells were randomly chosen, and the number of cells losing GFP-TMCC3 puncta was counted. The ratio of the cells losing GFP-TMCC3 puncta or GFP-TMCC3-S15A puncta to the transfected cells is expressed as a percentage and shown in the *bottom panel*. The error bars represent SD of six independent experiments. Statistical analysis was performed using Student’s *t* test. ∗∗∗*p* < 0.001. TMCC3, transmembrane and coiled-coil domain family 3.

We generated the full-length TMCC3 mutant substituting alanine for serine 15 (TMCC3-S15A) and established U2OS cell lines (U2OS-GFP-TMCC3-S15A cells) stably expressing GFP-tagged TMCC3-S15A (GFP-TMCC3-S15A). Immunoblotting of the total cell lysates showed that expression level of GFP-TMCC3-S15A in the U2OS-GFP-TMCC3-S15A cells was comparable to that of GFP-TMCC3 in the U2OS-GFP-TMCC3 cells used in Figure 1 (Fig. S4*A*). Immunostaining of the U2OS-GFP-TMCC3-S15A cells for PDI showed that GFP-TMCC3-S15A localized at three-way junctions (Fig. S4*B*). HA-14-3-3γ or a control vector was transfected into the U2OS-GFP-TMCC3 cells and U2OS-GFP-TMCC3-S15A cells, followed by immunostaining with the anti-HA mAb. Consistent with the results in Figure 1, overexpression of HA-14-3-3γ significantly increased the number of the U2OS-GFP-TMCC3 cells losing GFP-TMCC3 puncta relative to transfection of the control vector (Fig. 5). Overexpression of HA-14-3-3γ also increased the number of the U2OS-GFP-TMCC3-S15A cells losing GFP-TMCC3-S15A puncta relative to transfection of the control vector. However, the number of the cells losing GFP-TMCC3-S15A puncta was significantly lower than that of the cells losing GFP-TMCC3 puncta (Fig. 5), indicating that TMCC3-S15A was prone to localize at three-way junctions against overexpressed 14-3-3γ.

Collectively, these results indicate that 14-3-3γ negatively regulates localization of TMCC3 to the three-way junctions through binding to phosphoserine 15.

### The negative regulation of TMCC3 by 14-3-3γ is involved in formation of the reticular ER network

**Figure 6:**
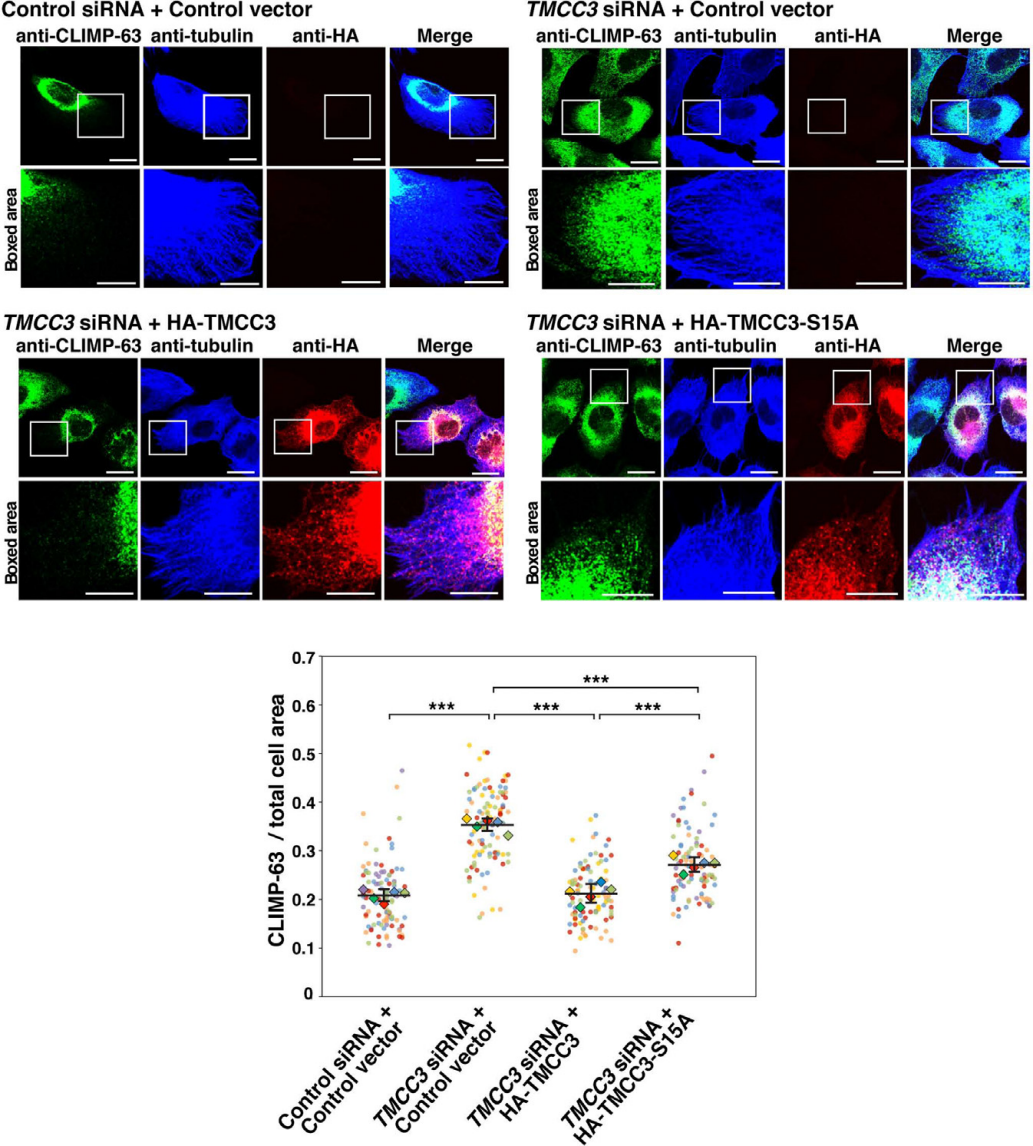
**The negative regulation of TMCC3 by 14-3-3γ is involved in formation of the reticular ER network**. The siRNA targeting TMCC3 was transfected into U2OS cells to allow endogenous TMCC3 to be knocked down. HA-TMCC3, HA-TMCC3-S15A, or the control vector was then transfected to the TMCC3-knockdown cells. As a negative control, the control siRNA was transfected into U2OS cells, followed by transfection of the control vector. The samples were subjected to immunostaining with the anti-HA mAb, the anti-CLIMP-63 pAb, and the anti-α-tubulin mAb. Representative images of five independent experiments are shown in the top and middle panels. Scale bars, 20 μm. The *boxed areas* are enlarged to highlight the ER sheet expansion and shown below each image. Scale bars, 10 μm. Twenty transfected cells were randomly chosen, and the pixel areas of CLIMP-63 and the total cell were measured in each cell. The ratio of CLIMP-63 area to the total cell area is shown in the bottom panel. Each dot indicates the ratio of CLIMP-63 area to the total cell area of a single cell. The five independent experiments are indicated by different colors. The mean value from each experiment is shown with rhombus. The bars represent mean ± SD from the five independent experiments. Statistical analysis was performed using Student’s *t* test. ∗∗∗*p* < 0.001. ER, endoplasmic reticulum; TMCC3, transmembrane and coiled-coil domain family 3.

We finally sought to assess the physiological importance of the negative regulation of TMCC3 by 14-3-3γ. To this end, we examined whether TMCC3-S15A could rescue the phenotype of TMCC3-knockdown cells. We had previously demonstrated that TMCC3 knockdown caused the ER sheet expansion (40). The siRNA targeting TMCC3 was transfected into U2OS cells to allow endogenous TMCC3 to be knocked down. Knockdown of endogenous TMCC3 was confirmed by immunoblotting (Fig. S5). The TMCC3-knockdown cells were then transfected with HA-TMCC3, HA-TMCC3-S15A, or the control vector. It is noted that HA-TMCC3 and HA-TMCC3-S15A are from mouse *TMCC3*, and resistant to the siRNA targeting human *TMCC3*, because mouse *TMCC3* intrinsically have the silent mutations within the siRNA target sequence of human *TMCC3*. As a negative control, the control siRNA was transfected into U2OS cells, followed by transfection of the control vector. The samples were subjected to immunostaining with the anti-HA mAb, the anti-CLIMP-63 pAb, and the anti-α-tubulin mAb, followed by quantification of abundance of the ER sheets. Note that CLIMP-63 is an ER sheet marker protein, and that α-tubulin staining allows us to determine outlines of the transfected cells. The negative control cells restricted CLIMP-63 localization predominantly to the perinuclear area where the ER sheets were enriched (Fig. 6). By contrast, TMCC3-knockdown cells transfected with the control vector extended CLIMP-63 localization to the peripheral area. The ratio of CLIMP-63 staining area to total area as judged by α-tubulin staining was significantly increased in the TMCC3-knockdown cells transfected with the control vector relative to the negative control cells (Fig. 6), indicating that, consistent with our previous finding (40), TMCC3 knockdown caused ER sheet expansion. On the other hand, the TMCC3-knockdown cells expressing HA-TMCC3 showed the ratio of CLIMP-63 staining area to total area comparable to the negative control cells, indicating that expression of HA-TMCC3 rescued the phenotype of the TMCC3 knockdown. Importantly, the TMCC3-knockdown cells expressing HA-TMCC3-S15A showed the ratio of CLIMP-63 staining area to total area significantly higher than the TMCC3-knockdown cells expressing HA-TMCC3, but lower than the TMCC3-knockdown cells transfected with the control vector. In the TMCC3-knockdown cells, HA-TMCC3-S15A showed puncta-like localization comparable to HA-TMCC3. These results indicate that expression of HA-TMCC3-S15A partially rescued the phenotype of the TMCC3 knockdown, suggesting that the negative regulation of TMCC3 by 14-3-3γ was involved in formation of the reticular ER network.

## Discussion

In this study, we demonstrate that 14-3-3γ binds to TMCC3 in a phosphorylation-dependent manner and negatively regulates localization of TMCC3 to the three-way junctions. Overexpression of 14-3-3γ reduces localization of TMCC3 to three-way junctions and decreased the number of three-way junctions, which is in agreement with the phenotype of TMCC3-knockdown cells. These results suggest that the negative regulation of TMCC3 by 14-3-3γ has the potency to regulate the reticular ER network. Of note, TMCC3-S15A, the mutant reducing the binding to 14-3-3γ, cannot fully rescue the phenotype of TMCC3-knockdown cells as shown in Figure 6, suggesting that the negative regulation of TMCC3 by 14-3-3γ is indeed important for formation of the reticular ER network *in vivo*. However, the detailed molecular mechanisms of how 14-3-3γ negatively regulate TMCC3 for the reticular ER network still remains unclear.

Overexpression of 14-3-3γ alters the peripheral ER morphology as shown in Figures 2 and S2. Since TMCC3 is the regulator for the ER morphology (40), we think that the morphological change of the ER caused by overexpression of 14-3-3γ would be mostly attributed to displacement of TMCC3 from three-way junctions. However, we cannot rule out the possibility that 14-3-3γ might also target as-yet-unknown proteins other than TMCC3, thereby contributing morphological change of the ER to some extent. Further studies will be required to address the effects of overexpression of 14-3-3γ on the ER morphology.

We have previously demonstrated that TMCC3 requires the first coiled-coil domain in the N-terminal cytoplasmic region for localization of three-way junctions (40). Therefore, one possible way for 14-3-3γ to inhibit localization of TMCC3 would be inactivation of the first coiled-coil domain by binding. Coiled-coils have been accepted as protein-protein interaction domain (58), although we do not know whether there exists a protein(s) that binds to the first coiled-coil domain. Given that the 14-3-3 binding motif is close to the first coiled-coil domain, there is the possibility that binding of 14-3-3γ to TMCC3 might interfere with interaction between TMCC3 and the possible binding-protein(s) that promotes localization of TMCC3. Another possibility is that 14-3-3γ would mask the first coiled-coil domain by binding. TMCC3-D1-S15/25/46A still showed weak binding to 14-3-3γ as shown in Figure 4, raising the possibility that there might exist a secondary 14-3-3 binding motif in the C-terminal part of TMCC3-D1. 14-3-3 Pred also predicted that serine 166 and threonine 176 would be additional potential binding sites for 14-3-3 proteins, although they were predicted with much lower scores than serine 15. In agreement with the low scores, both serine 166 and threonine 176 do not meet the consensus binding motif (RXXpS/TXP) (Fig. S6). While we do not know whether serine 166 and threonine 176 are phosphorylated *in vivo*, we cannot rule out the possibility that these amino acids might be additional binding sites for 14-3-3 proteins. Interestingly, both serine 166 and threonine 176 are behind the first coiled-coil domain (Fig. S6). It is well established that 14-3-3 proteins exist as a dimer in cytosol (47, 48, 49). Therefore, 14-3-3 dimers might bind to both serine 15 and serine 166 or threonine 176, thereby masking the first coiled–coil domain for negative regulation of localization of TMCC3. Further studies will be required to address these concerns.

Binding of 14-3-3γ to TMCC3 depends on phosphorylation of serine 15 in the 14-3-3 binding motif as shown in Figure 4, suggesting that regulation of phosphorylation of TMCC3 is important for the reticular ER morphology. Phosphorylation of serine 15 was detected in both HEK293 cells and U2OS cells as shown in Figures 4 and S3. In addition, the previous proteomic study demonstrated that TMCC3 is phosphorylated at serine 15 in adipocyte (59). Therefore, phosphorylation of serine 15 will not be a cell-type–specific effect. However, what kinase phosphorylates serine 15 of TMCC3 remains to be identified. Extracellular signals and cellular stresses are well known to elicit phosphorylation cascades mediated by kinases, and evidence is accumulating that 14-3-3 proteins bind to various proteins in the phosphorylation cascades (50, 51, 52, 53, 54, 55). For instance, TSC2 and PRAS40, two distinct regulators of mTORC1, are phosphorylated by AKT in response to insulin and bind to 14-3-3 proteins, which in turn prevents inactivation of mTORC1, leading to promotion of anabolic metabolism (60, 61, 62). Cdc25B and Cdc25C, protein phosphatase mediating cell cycle progression, are phosphorylated by Chk1 in response to DNA damage and binds to 14-3-3 proteins, which in turn prevent dephosphorylation of Cdk1, leading to G_2_ arrest (63, 64, 65). These evidences raise the possibility that binding of 14-3-3γ to TMCC3 might be also regulated by extracellular signals and/or cellular stresses. In our preliminary Co-IP analysis, serum depletion and an ER stressor did not affect binding of 14-3-3γ to TMCC3-D1 (data not shown). We, furthermore, observed that nocodazole, an inhibitor of mitosis, did not affect phosphorylation of TMCC3-D1 (Fig. S7), suggesting that mitosis will not be involved in phosphorylation of TMCC3. Therefore, TMCC3 might be in the phosphorylation cascades other than growth factor signaling, ER stress response, and mitosis. On the other hand, when we transfected exogenous TMCC3 into HEK293 cells and U2OS cells, phosphorylation of serine 15 was detected as shown in Figures 4 and S3, suggesting that the as-yet-unknown kinase phosphorylates TMCC3 even under normal conditions. Nevertheless, GFP-TMCC3 mostly localizes at three-way junctions in the cell line stably expressing GFP-TMCC3 under normal conditions as shown in Figures 1 and S1. These conflicting results presumably suggest that serine 15 in TMCC3 would undergo cycles of phosphorylation and dephosphorylation. In this case, the phosphorylation and dephosphorylation activities would be spatially and temporally controlled for the reticular ER network. The dephosphorylation activity might be stronger in the three-way junctions than the ER tubules, whereas the phosphorylation activity might be stronger in the ER tubules than the three-way junctions.

Phosphorylation of TMCC3-D1 was detected in both HEK293 and U2OS cells as shown in Figures 4 and S3. However, the immunoreactive band of phosphorylated HA-TMCC3-D1 was weaker than that of total HA-TMCC3-D1. While endogenous 14-3-3 was coimmunoprecipitated with GFP-TMCC3 from U2OS cells stably expressing GFP-TMCC3 as shown in Figure 3, we could not detect coimmunoprecipitation of endogenous 14-3-3 with endogenous TMCC3 from parental U2OS cells (data not shown). We, therefore, expect that the constitutive phosphorylation of TMCC3-D1 as detected in both HEK293 and U2OS cells will be presumably due to overexpression. A small population of endogenous TMCC3 seems most likely to be transiently phosphorylated at serine 15 under the normal condition. However, given that TMCC3-S15A cannot fully rescue the phenotype of TMCC3-knockdown cells as shown in Figure 6, this transient phosphorylation of TMCC3 will be important for the establishment and maintenance of the reticular ER morphology.

It remains unclear why TMCC3 must be negatively regulated for formation of the reticular ER network. Since TMCC3 acts as an upstream regulator of atlastins for formation of the three-way junctions (40), it is easy to understand that TMCC3 is required for the reticular ER network. In this context, the negative regulation of TMCC3 by 14-3-3γ would be expected to inhibit formation of the three-way junctions, leading to disruption of the reticular ER network. Indeed, overexpression of 14-3-3γ decreased the number of the three-way junctions as shown in Figure 2. Nevertheless, the partial rescue of the TMCC3-knockdown cells by TMCC3-S15A, as shown in Figure 6, suggests that the negative regulation of TMCC3 by 14-3-3γ is also involved in formation of the reticular ER network. Given that the reticular ER network is not static but undergoes constant remodeling through formation and loss of the three-way junctions (28, 29, 43, 44, 45), regulation of the atlastin activity is likely to underlie remodeling of the reticular ER network. Therefore, one possible explanation is that the negative regulation of TMCC3 by 14-3-3γ would contribute to remodeling of the reticular ER network through spatial and temporal regulation of the membrane fusogenic activity of atlastins. To further assess roles of the negative regulation of TMCC3 by 14-3-3γ, we introduced phosphomimetic mutations into serine 15 (TMCC3-S15D and TMCC3-S15E). However, TMCC3-S15D and TMCC3-S15E did not act as phosphomimetic mutants for 14-3-3 binding, because they did not enhance binding to 14-3-3γ and still localized at three-way junctions (data not shown). We do not think that these suggest inconsistency in our results, because the previous study of 14-3-3 binding sites states that glutamate and aspartate do not provide good phosphomimetic residues with respect to 14-3-3 binding to target proteins (66). Further studies will be required to address these concerns.

In summary, we demonstrate that 14-3-3γ binding negatively regulates localization of TMCC3 for the reticular ER network.

## Experimental procedures

### Antibodies

The antibodies were purchased from the following commercial sources: mouse anti-HA mAb (BioLegend; Cat. No. 901514), rabbit anti-HA pAb (Sigma-Aldrich; Cat. No. H6908), rat anti-HA mAb (Roche; Cat. No. 11867423001), mouse anti-FLAG mAb (Sigma-Aldrich; Cat. No. F1804), rabbit anti-FLAG pAb (Sigma-Aldrich; Cat. No. F7425), rabbit anti-TMCC3 pAb (Sigma-Aldrich; Cat. No. HPA014272), rabbit anti-CLIMP-63 pAb (Bethyl Laboratories; Cat. No. A302–257A), mouse anti-α-tubulin mAb (Sigma-Aldrich; Cat. No. T6199), mouse anti-14-3-3 mAb (Santa Cruz Biotechnology; Cat. No. sc-1657), rabbit anti-phospho 14-3-3 binding motif pAb (Cell Signaling; Cat. No. 9601), mouse anti-PDI mAb (Abcam; Cat. No. ab2792), rat anti-GFP mAb (Nacalai; Cat. No. 04404–84), rabbit anti-GFP pAb (MBL; Cat. No. 598), and mouse anti-GAPDH mAb-HRP-DirecT (MBL; Cat. No. M171–7).

### Plasmids

The cDNA encoding full-length mouse TMCC3 and the cDNAs encoding the following fragments of mouse TMCC3 [1 to 411 aa (TMCC3-N); 391 to 477 aa (TMCC3-C); 1 to 210 aa (TMCC3-D1); 90 to 210 aa (TMCC3-D2)] were subcloned into the pCMV vector with the N-terminal HA tags. pCMV-HA-TMCC3 and pCMV-HA-TMCC3-D1 were subjected to site-directed mutagenesis to generate the TMCC3 mutants substituting alanine for serine 15 (TMCC3-S15A and TMCC3-D1-S15A, respectively). pCMV-HA-TMCC3-D1-S15A was subjected to the second round of site-directed mutagenesis to generate the TMCC3 mutant substituting alanines for serine 15, serine 25, and serine 46 (TMCC3-D1-S15/25/46A). The cDNA encoding mouse 14-3-3γ was subcloned into the pCMV vector with the N-terminal HA or Flag tag. For stable transfection, full-length TMCC3 and TMCC3-S15A were first subcloned into the pEGFP-C1 vector to generate GFP-TMCC3 and GFP-TMCC3-S15A. GFP-TMCC3 and GFP-TMCC3-S15A were then subcloned into the pCAGIpuro vector.

### Cell culture

HEK293 cells were maintained in Dulbecco’s modified Eagle’s medium (DMEM)/F12 (Gibco) supplemented with 5% FBS, penicillin, and streptomycin at 37 ˚C and 5% CO_2_. U2OS cells were maintained in DMEM (Nacalai Tesque) supplemented with 10% FBS, penicillin, and streptomycin at 37 °C and 5% CO_2_.

To establish U2OS cell lines stably expressing GFP-TMCC3 (U2OS-GFP-TMCC3 cells) or GFP-TMCC3-S15A (U2OS-GFP-TMCC3-S15A cells), pCAGIpuro-GFP-TMCC3, or pCAGIpuro-GFP-TMCC3-S15A was transfected into U2OS cells with Effectene (QIAGEN). The cells were cultured for 2 days, replated, and selected by culturing in the presence of 3 μg/ml puromycin (Sigma-Aldrich). The puromycin-resistant colonies were isolated and maintained in DMEM supplemented with 10% FBS, penicillin, streptomycin, and 3 μg/ml puromycin at 37 °C and 5% CO_2_.

### Immunohistochemistry

U2OS cells, U2OS-GFP-TMCC3 cells, and U2OS-GFP-TMCC3-S15A cells were cultured on coverslips, fixed with 4% paraformaldehyde, and permeabilized with 100 μg/ml digitonin except that 0.2% Triton X-100 was used for detection of PDI and CLIMP-63. After being blocked with PBS containing with 1% BSA, the samples were incubated with primary Abs, followed by incubation with secondary Abs conjugated with Alexa Fluor dyes (Invitrogen). After being washed with PBS, they were embedded and viewed using a confocal imaging system (ZEISS, LSM 510 Meta). In some instances, appropriate combinations of the plasmids were transfected into U2OS cells, U2OS-GFP-TMCC3 cells, and U2OS-GFP-TMCC3-S15A cells with Effectene (QIAGEN). One day or 6 h after transfection, the cells were subjected to immunostaining as described above.

### Immunoprecipitation

For coimmunoprecipitation of exogenous TMCC3 and exogenous 14-3-3γ, appropriate combinations of the mammalian expression vectors were transfected into HEK293 cells using Effectene (QIAGEN) or Lipofectamine LTX (Invitrogen) in accordance with the manufacturer’s manual. The day after transfection, the cells were lysed in buffer A (20 mM Tris–HCl pH7.5, 150 mM NaCl, 2 mM MgCl_2_, and 1% Triton X-100) supplemented with the protease inhibitors (10 μM APMSF, 10 μg/ml leupeptin, and 5 μg/ml aprotinin) at 4 °C for 30 min, followed by ultracentrifugation at 100,000*g* at 4 °C for 30 min. The Triton X-100 extracts were incubated with the mouse anti-Flag mAb or the mouse anti-HA mAb, followed by immunoprecipitation with Protein G Sepharose (GE Healthcare). The samples were subjected to SDS-PAGE followed by immunoblotting.

For coimmunoprecipitation of exogenous TMCC3 and endogenous 14-3-3 proteins, U2OS-GFP-TMCC3 cells were lysed in buffer B (20 mM Tris–HCl pH7.5, 150 mM NaCl, 1 mM EDTA, and 1% Triton X-100) supplemented with the protease inhibitors and the phosphatase inhibitor cocktails (Sigma-Aldrich; Cat. No. P0044) and ultracentrifuged in the same manner as described above. The Triton X-100 extracts of the U2OS cells were incubated with the rabbit anti-GFP pAb or rabbit control IgG at 4 °C for 3 h, followed by immunoprecipitation with Protein G Sepharose (GE Healthcare). The samples were subjected to SDS-PAGE followed by immunoblotting with the rat anti-GFP mAb and the anti-14-3-3 mAb.

For detection of phosphorylation of TMCC3, HEK293 cells transfected with HA-TMCC3-D1, or its mutants were lysed in Buffer A supplemented with the protease inhibitors and the phosphatase inhibitor cocktail, and the Triton X-100 extracts were subjected to immunoprecipitation with the mouse anti-HA mAb as described above.

### siRNA knockdown

U2OS cells were transfected with 10 μM of the silencer-select siRNAs targeting human *TMCC3* (Thermo Fisher Scientific; Cat. No. 4427037, ID s33058) or the control siRNA (Thermo Fisher Scientific; Cat. No. AM4613) using Lipofectamine RNAi MAX (Invitrogen) in accordance with the manufacturer’s manual. The cells were cultured for 2 days to allow endogenous TMCC3 to be depleted.

### Quantification of the ER morphology

As for quantification of three-way junctions, U2OS cells were transfected with pCMV-HA-14-3-3γ or pCMV-mCherry using Effectene. The day after transfection, the cells were fixed with 4% paraformaldehyde and permeabilized with 0.2% TritonX-100, followed by immunostaining with the anti-PDI mAb and the anti-HA mAb. 10 × 10 μm^2^ of the peripheral ER area was randomly chosen in each of the transfected cells, and the number of three-way junctions as detected by PDI staining was counted. In some instances, HA-14-3-3γ or the control vector was transfected into U2OS cells. The day after transfection, the cells were further transfected with ER-GFP using the recombinant baculovirus harboring ER-GFP (CellLight ER-GFP, BacMam 2.0, Thermo Fisher) in accordance with the manufacturer’s manual. The cells were then immunostained with the anti-HA mAb and the anti-GFP mAb as described above, and the number of three-way junctions as detected by ER-GFP within 10 × 10 μm^2^ of the peripheral ER was counted.

As for quantification of ER sheets, the TMCC3-knockdown cells were transfected with pCMV-HA-TMCC3, pCMV-HA-TMCC3-S15A, or the pCMV-HA vector alone and further cultured for 24 h to allow expression of HA-TMCC3 or HA-TMCC3-S15A. The cells were fixed with 4% paraformaldehyde and permeabilized with 0.2% Triton X-100, followed by immunostaining with the anti-CLIMP-63 pAb, the anti-HA mAb, and the anti-α-tubulin mAb. The images of CLIMP-63 staining were converted into binary images using Image J, and the pixel area of CLIMP-63 staining in each cell was measured. The outline of each cell was determined by α-tubulin staining, and the pixel area of the total cell was measured with Image J. Then, the ratio of CLIMP-63 to the total cell area was calculated for each cell.

## Data availability

All data were contained within the article.

## Supporting information

This article contains supporting information.

## Conflict of interest

The authors declare no conflicts of interest with the contents of the article.

## Supporting information

Figure S1 **Characterization of U2OS cells stably expressing GFP-TMCC3**. The U2OS-GFP-TMCC3 cells were immunostained with the anti-PDI mAb. GFP-TMCC3 was detected by its fluorescence. The *boxed areas* are enlarged to highlight the peripheral ER and shown below each image. *Arrowheads* indicate localization of GFP-TMCC3 at three-way junctions. Scale bars, 20 μm. TMCC3, transmembrane and coiled-coil domain family 3; PDI, protein disulfide isomerase; ER, endoplasmic reticulum.

Figure S2 **Effects of overexpression of 14-3-3γ on the ER morphology.** HA-14-3-3γ or the control vector was transfected into U2OS cells, followed by transfection with ER-GFP using the recombinant baculovirus carrying ER-GFP (CellLight™ ER-GFP, BacMam 2.0, Thermo Fisher Scientific). The cells were permeabilized with Triton X-100, and immunostained with the anti-HA mAb, and the anti-GFP mAb. Forty-eight transfected cells were analyzed, and the representative images are shown in the left panels. Scale bars, 20 μm. The *boxed areas* represent 10 × 10 μm^2^ of the peripheral ER and enlarged in the lower panels. *Arrowheads* indicate representatives of the three-way junctions. Scale bars, 5 μm. The number of three way junctions as detected by ER-GFP was counted within 10 × 10 μm^2^ of the peripheral ER. The number of three-way junctions per cell is shown in the right graph. Each *dot* represents an individual cell. The bars represent mean ± SD. Statistical analysis was performed using Student’s *t*-test. ∗∗∗*P*<0.001. ER, endoplasmic reticulum.

Figure S3 P**hosphorylation of serine 15 in U2OS cells**. HA-TMCC3-D1 and HA-TMCC3-D1-S15A were transfected into U2OS cells, followed by immunoprecipitation with the anti-HA mAb. The samples were immunoblotted with the anti-phospho-14-3-3 binding motif pAb and the anti-HA pAb. The *asterisk* indicates the nonspecific bands from the light chain of IgG. TMCC3, transmembrane and coiled-coil domain family 3.

Figure S4 **Characterization of U2OS cells stably expressing GFP-TMCC3-S15A.***A*, checking the expression level of GFP-TMCC3-S15A. Total cell lysates (5 μg) of U2OS-GFP-TMCC3 cells and U2OS-GFP-TMCC3-S15A cells were subjected to SDS-PAGE followed by immunoblotting with the anti-GFP mAb and anti-GAPDH mAb. *B*, localization of GFP-TMCC3-S15A at three-way junctions. The U2OS-GFP-TMCC3-S15A cells were immunostained with the anti-PDI mAb. GFP-TMCC3-S15A was detected by its fluorescence. Scale bar, 20 μm. The *boxed areas* are enlarged to highlight the peripheral ER and shown below each image. *Arrowheads* indicate localization of GFP-TMCC3-S15A at three-way junctions. Scale bar, 10 μm. TMCC3, transmembrane and coiled-coil domain family 3; ER, endoplasmic reticulum; PDI, protein disulfide isomerase.

Figure S5 **Knockdown of endogenous TMCC3 by siRNA**. U2OS cells were transfected with the control siRNA or *TMCC3* siRNA. The total cell lysates were subjected to immunoblotting with the anti-TMCC3 pAb and the anti-GAPDH mAb. The *arrowhead* indicates endogenous TMCC3. The immunoreactive band of endogenous TMCC3 was significantly decreased in *TMCC3* siRNA-transfected cells, indicating that endogenous TMCC3 was efficiently knocked down. TMCC 3, transmembrane and coiled-coil domain family 3.

Figure S6 **Additional potential binding sites for 14-3-3 in TMCC3-D1**. The sequence shown here is the N-terminal 210 aa of mouse TMCC3 (TMCC3-D1). Serine 166 and threonine 176, additional potential binding sites for 14-3-3 proteins which are predicted by 14-3-3 Pred with much lower scores than serine 15, are shown in *green*. Serine 15 that we characterized in this study is shown in *red*. The first coiled-coil domain is underlined. TMCC3, transmembrane and coiled-coil domain family 3.

Figure S7 **No effect of nocodazole treatment on phosphorylation of serine 15**. HA-TMCC3-D1 or HA-TMCC3-D1-S15A was transfected into HEK293 cells. The day after transfection, the culture medium was replaced with fresh one supplemented with 100 ng/mL nocodazole or DMSO as a negative control, and the cells were further cultured for 17 h. The cells were subjected to immunoprecipitation with the anti-HA mAb, followed by immunoblotting with the anti-phospho-14-3-3 binding motif pAb and the anti-HA mAb. The anti-phospho-14-3-3 binding motif pAb detected HA-TMCC3-D1 but did not recognize HA-TMCC3-D1-S15A, while the anti-HA mAb detected HA-TMCC3-D1 and HA-TMCC3-D1-S15A. Importantly, the immunoreactive band of the phosphorylated HA-TMCC3-D1 in the presence of DMSO was comparable to that in the presence of nocodazole, suggesting that mitosis will not affect the phosphorylation status of TMCC3. TMCC3, transmembrane and coiled-coil domain family 3.
