# Supplementary figures and images for: The 14-3-3γ isoform binds to and regulates the localization of endoplasmic reticulum (ER) membrane protein TMCC3 for the reticular network of the ER
Source: J Biol Chem. 2022 Dec 20;299(2):102813. doi: 10.1016/j.jbc.2022.102813 (PMC9860497; doi:10.1016/j.jbc.2022.102813)

**Figure S1**

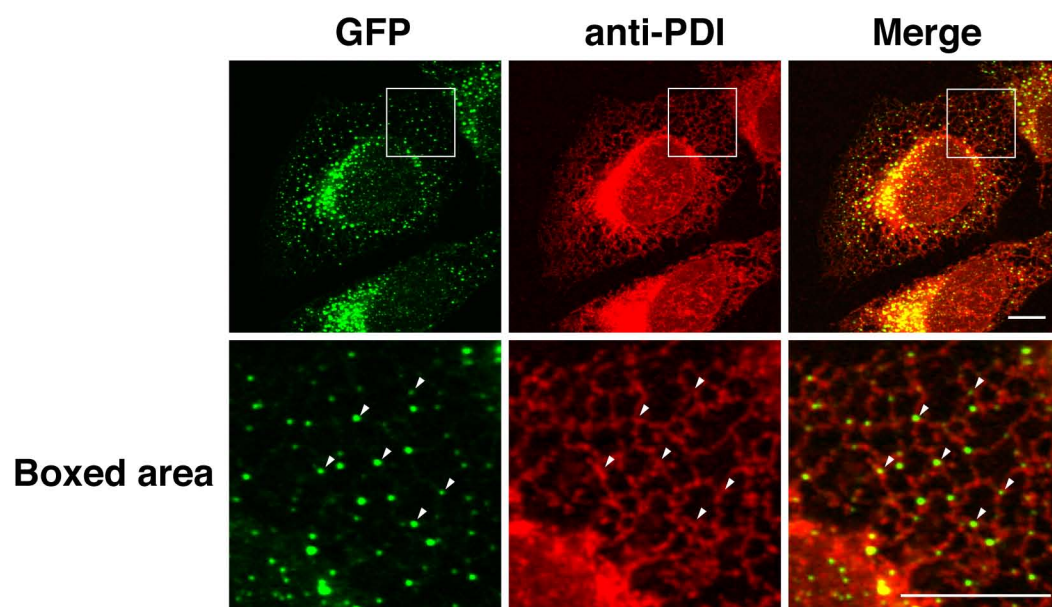

Supplement: Figure S1 — Characterization of U2OS cells stably expressing GFP-TMCC3. The U2OS-GFP-TMCC3 cells were immunostained with the anti-PDI mAb. GFP-TMCC3 was detected by its fluorescence. The boxed areas are enlarged to highlight the peripheral ER and shown below each image. Arrowheads indicate localization of GFP-TMCC3 at three-way junctions. Scale bars, 20 μm. TMCC3, transmembrane and coiled-coil domain family 3; PDI, protein disulfide isomerase; ER, endoplasmic reticulum. [file mmc1.pdf]

# Figure S2

U2OS cells + control vector + ER-GFP

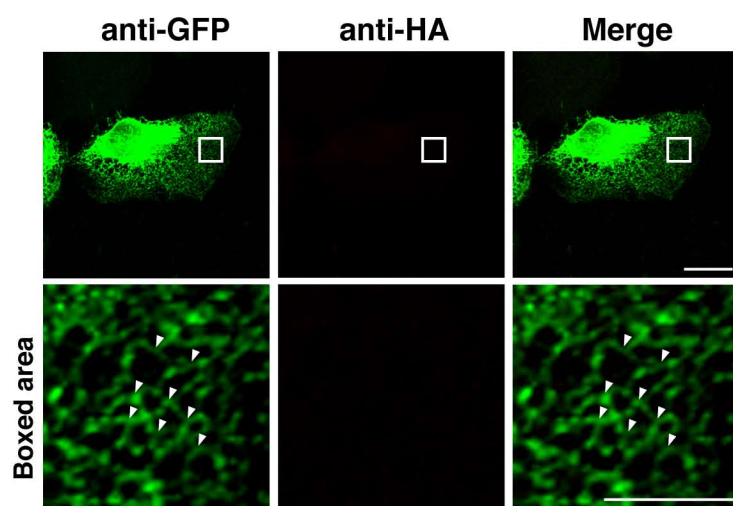

U2OS cells + HA-14-3-3 $\gamma$  + ER-GFP

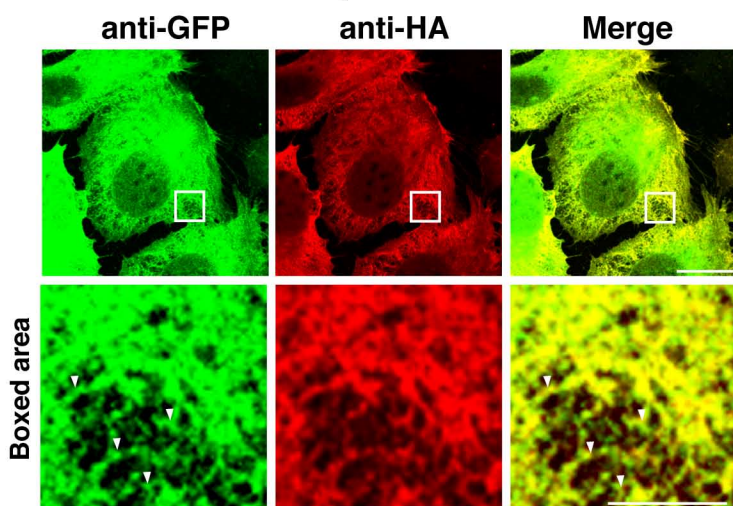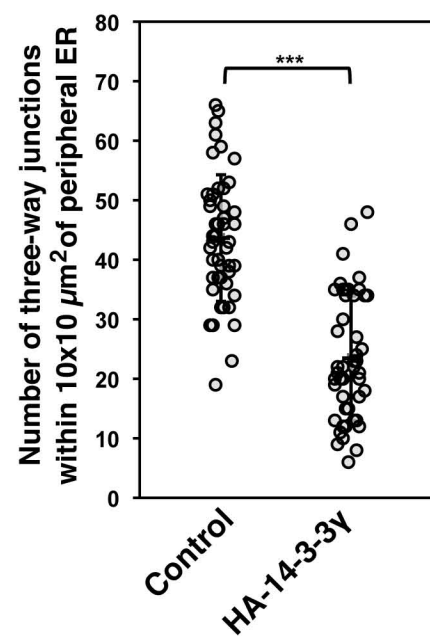

Supplement: Figure S2 — Effects of overexpression of 14-3-3γ on the ER morphology. HA-14-3-3γ or the control vector was transfected into U2OS cells, followed by transfection with ER-GFP using the recombinant baculovirus carrying ER-GFP (CellLight™ ER-GFP, BacMam 2.0, Thermo Fisher Scientific). The cells were permeabilized with Triton X-100, and immunostained with the anti-HA mAb, and the anti-GFP mAb. Forty-eight transfected cells were analyzed, and the representative images are shown in the left panels. Scale bars, 20 μm. The boxed areas represent 10 × 10 μm2 of the peripheral ER and enlarged in the lower panels. Arrowheads indicate representatives of the three-way junctions. Scale bars, 5 μm. The number of three way junctions as detected by ER-GFP was counted within 10 × 10 μm2 of the peripheral ER. The number of three-way junctions per cell is shown in the right graph. Each dot represents an individual cell. The bars represent mean ± SD. Statistical analysis was performed using Student’s t-test. ∗∗∗P<0.001. ER, endoplasmic reticulum. [file mmc2.pdf]

Figure S3

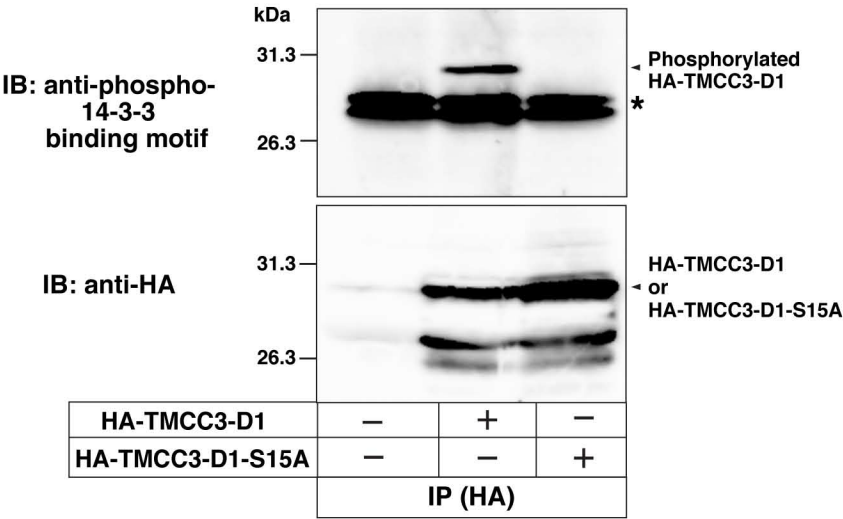

Supplement: Figure S3 — Phosphorylation of serine 15 in U2OS cells. HA-TMCC3-D1 and HA-TMCC3-D1-S15A were transfected into U2OS cells, followed by immunoprecipitation with the anti-HA mAb. The samples were immunoblotted with the anti-phospho-14-3-3 binding motif pAb and the anti-HA pAb. The asterisk indicates the nonspecific bands from the light chain of IgG. TMCC3, transmembrane and coiled-coil domain family 3. [file mmc3.pdf]

Figure S4

**A**

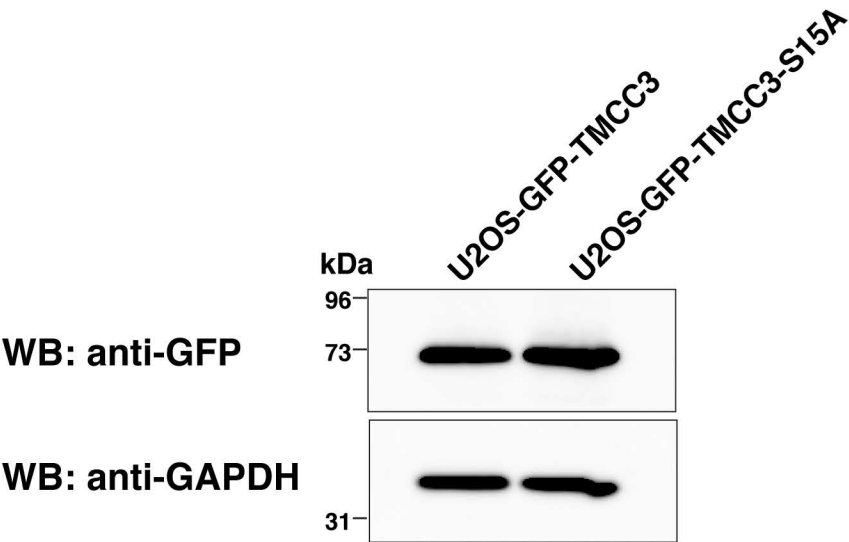

**B**

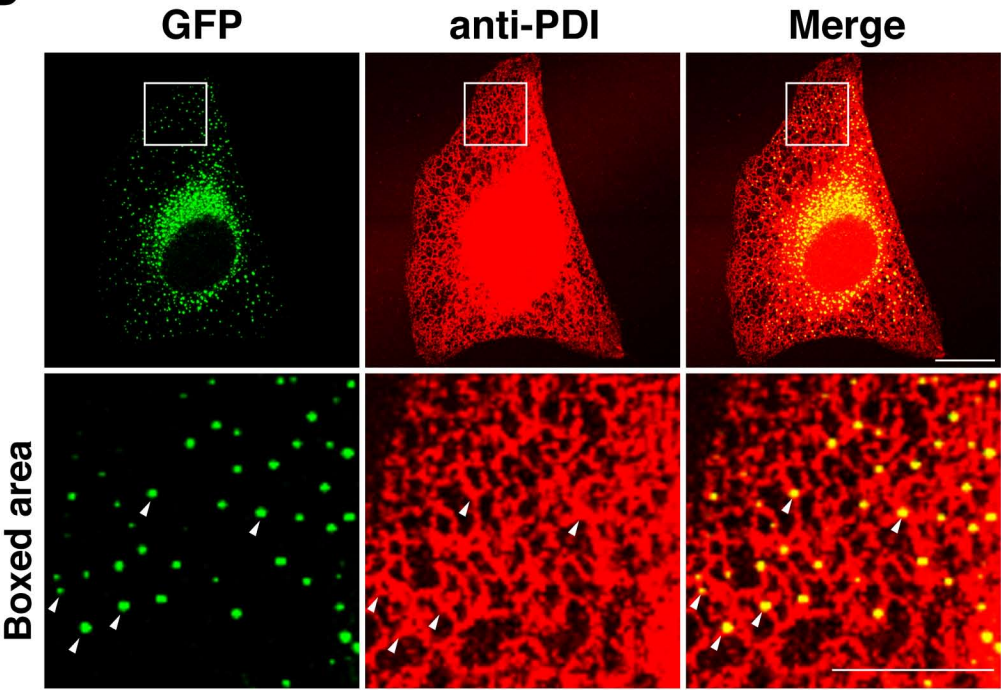

Supplement: Figure S4 — Characterization of U2OS cells stably expressing GFP-TMCC3-S15A.A, checking the expression level of GFP-TMCC3-S15A. Total cell lysates (5 μg) of U2OS-GFP-TMCC3 cells and U2OS-GFP-TMCC3-S15A cells were subjected to SDS-PAGE followed by immunoblotting with the anti-GFP mAb and anti-GAPDH mAb. B, localization of GFP-TMCC3-S15A at three-way junctions. The U2OS-GFP-TMCC3-S15A cells were immunostained with the anti-PDI mAb. GFP-TMCC3-S15A was detected by its fluorescence. Scale bar, 20 μm. The boxed areas are enlarged to highlight the peripheral ER and shown below each image. Arrowheads indicate localization of GFP-TMCC3-S15A at three-way junctions. Scale bar, 10 μm. TMCC3, transmembrane and coiled-coil domain family 3; ER, endoplasmic reticulum; PDI, protein disulfide isomerase. [file mmc4.pdf]

**Figure S5**

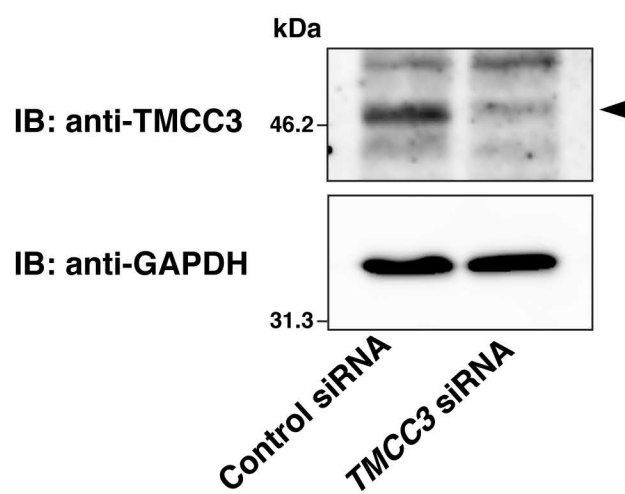

Supplement: Figure S5 — Knockdown of endogenous TMCC3 by siRNA. U2OS cells were transfected with the control siRNA or TMCC3 siRNA. The total cell lysates were subjected to immunoblotting with the anti-TMCC3 pAb and the anti-GAPDH mAb. The arrowhead indicates endogenous TMCC3. The immunoreactive band of endogenous TMCC3 was significantly decreased in TMCC3 siRNA-transfected cells, indicating that endogenous TMCC3 was efficiently knocked down. TMCC 3, transmembrane and coiled-coil domain family 3. [file mmc5.pdf]

Figure S7

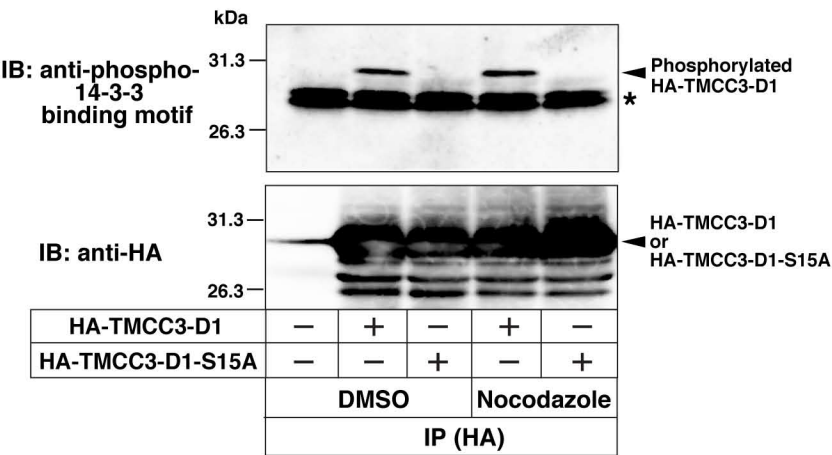

Supplement: Figure S7 — No effect of nocodazole treatment on phosphorylation of serine 15. HA-TMCC3-D1 or HA-TMCC3-D1-S15A was transfected into HEK293 cells. The day after transfection, the culture medium was replaced with fresh one supplemented with 100 ng/mL nocodazole or DMSO as a negative control, and the cells were further cultured for 17 h. The cells were subjected to immunoprecipitation with the anti-HA mAb, followed by immunoblotting with the anti-phospho-14-3-3 binding motif pAb and the anti-HA mAb. The anti-phospho-14-3-3 binding motif pAb detected HA-TMCC3-D1 but did not recognize HA-TMCC3-D1-S15A, while the anti-HA mAb detected HA-TMCC3-D1 and HA-TMCC3-D1-S15A. Importantly, the immunoreactive band of the phosphorylated HA-TMCC3-D1 in the presence of DMSO was comparable to that in the presence of nocodazole, suggesting that mitosis will not affect the phosphorylation status of TMCC3. TMCC3, transmembrane and coiled-coil domain family 3. [file mmc7.pdf]
