# Supplementary material for: The 14-3-3γ isoform binds to and regulates the localization of endoplasmic reticulum (ER) membrane protein TMCC3 for the reticular network of the ER
Source: J Biol Chem. 2022 Dec 20;299(2):102813. doi: 10.1016/j.jbc.2022.102813 (PMC9860497; doi:10.1016/j.jbc.2022.102813)
Supplement: Figure S6 — Additional potential binding sites for 14-3-3 in TMCC3-D1. The sequence shown here is the N-terminal 210 aa of mouse TMCC3 (TMCC3-D1). Serine 166 and threonine 176, additional potential binding sites for 14-3-3 proteins which are predicted by 14-3-3 Pred with much lower scores than serine 15, are shown in green. Serine 15 that we characterized in this study is shown in red. The first coiled-coil domain is underlined. TMCC3, transmembrane and coiled-coil domain family 3. [file mmc6.pdf]

Figure S6

1 MPGSDTALTVDRTYSDPGRHHRCKSRVDRHDMNTLSLPLNIRRGGSDTNL

51 NFDVPDGILDFHKVKLNADSLRQKILKVTEQIKIEQTSRDGNVAEYLKLV

101 SSADKQQAGRIKQVFEKKNQKSAHSIAQLQKKLEQYHRKLREIEQNGVTR

Coiled-coil 1

151 SSKDISKDSLKEIHHSLKDAHVKSRTAPHCLESSKSSMPGVSLTPPVFVF

201 NKSREFANLI
